# Exploring the molecular mechanism of EGCG in preventing obesity-induced precocious puberty based on serum metabolomics and molecular docking

**DOI:** 10.3389/fnut.2025.1675535

**Published:** 2025-11-06

**Authors:** Shiyu Gao, Lina Xia, Chenzhenghao Jiang, Bang Shao, Ying Shao, Xiaojing Li, Peiying Wu, Jieyi He, Qiujv Du, Lingwei Liang, Qiuyun Gu

**Affiliations:** 1 Department of Nutrition, Shanghai General Hospital, Shanghai Jiao Tong University School of Medicine, Shanghai, China; 2 Department of Clinical Nutrition, College of Health Science and Technology, Shanghai Jiao Tong University School of Medicine, Shanghai, China; 3 Department of Pharmacy, Women's Hospital of Nanjing Medical University, Nanjing Maternity and Child Health Care Hospital, Nanjing, China

**Keywords:** epigallocatechin gallate, obesity-induced precocious puberty, differential metabolites, serum metabolomics, molecular docking

## Abstract

**Objective:**

Obesity-induced precocious puberty presents serious health risks to adolescents. Building on our previous finding that epigallocatechin gallate (EGCG) exhibits a preventive effect on obesity-induced precocious puberty, the present study aims to elucidate the underlying molecular mechanisms.

**Methods:**

Female C57BL/6 mice were divided into four groups: control, normal diet + EGCG, high-fat diet (HFD), and HFD + EGCG. Body weight, vaginal opening time, and serum samples were analyzed to assess the effects of EGCG on obesity-induced precocious puberty, using serum metabolomics and molecular docking.

**Results:**

EGCG treatment significantly altered the serum metabolite profile, particularly affecting lipid metabolism. Glycerophospholipid metabolism emerged as the key pathway modulated by EGCG. Molecular docking identified phosphatidylserine decarboxylase, phospholipase D, and phosphatidylserine synthase as potential targets.

**Conclusion:**

EGCG prevents obesity-induced precocious puberty, an effect associated with the reshaping of lipid metabolism, with key enzymes in the glycerophospholipid metabolism serving as potential therapeutic targets. These findings provide a foundational hypothesis for further mechanistic investigation.

## 1 Introduction

Precocious puberty is clinically defined by the premature development of thelarche (breast budding) in girls under 8 years of age and gonadarche, specifically, a testicular volume exceeding 4 mL in boys under 9 years of age, which is accompanied by accelerated skeletal bone age advancement and rapid linear growth (1). Childhood precocious puberty is associated with psychological and social dysfunctions, including depression, mental health issues, and impaired social adaptation (2), and also poses long-term health risks such as reduced adult height, metabolic disorders (e.g., hypertension and type 2 diabetes), cardiovascular diseases (e.g., ischemic heart disease and stroke), and reproductive system malignancies (3). Therefore, this condition warrants significant public health attention. Implementing proactive measures to prevent and manage precocious puberty is crucial for ensuring the healthy physical and psychological development of adolescents and enhancing their long-term health outcomes.

The risk factors associated with precocious puberty are multifaceted, encompassing obesity, genetic predispositions, lifestyle habits, and environmental endocrine disruptors (4, 5). Recently, obesity-induced precocious puberty has emerged as a growing public health concern, particularly in developed nations where childhood obesity rates have markedly increased (6). The accumulation of adipose tissue can promote the onset of precocious puberty in children. The underlying mechanisms include hormonal imbalances, elevated leptin levels, and alterations in the hypothalamic–pituitary-gonadal axis (HPGA). Consequently, childhood obesity represents a well-established, significant risk factor for the premature onset of puberty. Thus, obesity prevention constitutes a critical strategy in the prevention and management of childhood precocious puberty (7, 8).

Metabolomics represents a high-throughput analytical field focused on the comprehensive quantification of small-molecule metabolites within biological systems (9). Its core methodology involves selecting relevant metabolites and comparing metabolic profiles across different physiological or pathological conditions, thereby revealing alterations in metabolic pathways that may underlie disease mechanisms or drug effects (10). Several comprehensive and systematic studies have employed metabolomic techniques to detect alterations in metabolite levels in girls with obesity-induced precocious puberty. For instance, Li et al. compared the serum samples of 50 normal girls and girls with central precocious puberty using liquid chromatography–tandem mass spectrometry (LC–MS/MS) and identified 103 differentially expressed metabolites (11). These metabolites were primarily enriched in linoleic acid metabolism, neuroactive ligand receptor interaction, and phospholipase D signaling pathway. Zhao et al. (12) compared serum metabolic profiles between 10 girls with central precocious puberty and age-matched female controls. The analysis revealed that the differential metabolites were significantly enriched in steroid hormone biosynthesis, bile secretion, histidine metabolism, and *β*-alanine metabolism. The above research has identified potential biomarkers and metabolic pathways associated with obesity-induced precocious puberty, thereby providing a theoretical basis for understanding the pathogenesis of sexual precocity.

The exact pathogenic mechanism underlying precocious puberty remains unclear; however, it has been established that the condition results from the premature activation of the HPGA. This activation leads to an increase in the secretion of gonadotropin-releasing hormone (GnRH) by the hypothalamus, which subsequently stimulates early gonadal development and the secretion of sex hormones. These processes result in the premature development of internal and external reproductive organs and the emergence of secondary sexual characteristics (13). Clinically, GnRH agonists (GnRHa) are the first-line treatment for central precocious puberty (14). They can inhibit the secretion of follicle-stimulating hormone and luteinizing hormone, thereby suppressing gonadal activity and counteracting precocious puberty. Although generally safe and well-tolerated in children (15), GnRHa therapy requires high doses, long treatment durations, and is associated with substantial costs. Adverse effects such as allergic reactions, vaginal bleeding, and sterile abscesses may cause discomfort and reduce treatment adherence among patients and their families (16, 17). Given these limitations, there is growing interest in exploring bioactive food components for preventing or mitigating precocious puberty.

Recently, research has explored the role of dietary components and specific bioactive compounds in modulating risk factors associated with precocious puberty. Epigallocatechin gallate (EGCG), a flavonoid found in tea, has shown promise in preventing obesity-induced precocious puberty (18, 19). As the most abundant and biologically active polyphenol in tea, accounting for approximately 68–69% of tea polyphenols (20), EGCG exhibits a range of beneficial properties, including anticancer, free-radical-scavenging, antioxidant, anti-inflammatory, hypoglycemic, and hypolipidemic effects (21, 22). Clinical studies have demonstrated that daily supplementation with 400 mg of green tea polyphenols for 3 months reduced body mass index and body fat percentage in obese girls, significantly decreased left ovarian volume, and helped prevent premature puberty (18). Animal studies have further indicated that EGCG delays vaginal opening in female rats fed a high-fat diet (HFD), supporting its protective role against diet-induced precocious puberty (19). Nonetheless, the molecular mechanisms underlying EGCG’s preventive effects on obesity-induced precocious puberty remain to be elucidated. While our previous research utilizing network pharmacology and multi-omics approaches has successfully identified the glycerophospholipid metabolism pathway as a key target for EGCG in the context of obesity-induced precocious puberty (23, 24), the specific molecular mechanisms by which EGCG modulates this pathway remained unexplored. In particular, the question of whether EGCG directly interacts with the core enzymes governing glycerophospholipid metabolism was unanswered. Therefore, the present study was designed to build upon these earlier findings by specifically investigating the direct binding potential of EGCG to key metabolic enzymes identified from our serum metabolomics analysis. We employed molecular docking simulations to bridge the gap between observed metabolic changes and potential protein targets, aiming to provide a more mechanistic understanding of EGCG’s action.

## 2 Methods

### 2.1 Experimental animals

In a controlled environment at the Animal Center of Shanghai General Hospital, 20 female C57BL/6 mice (21 days old; sourced from Shanghai SLAC Laboratory Animal Co., Ltd) were housed under standardized conditions, including a temperature of 22 ± 2 °C, a 12-h light/dark cycle, and access to food and water *ad libitum*. All experimental procedures were approved by the hospital’s Institutional Animal Care Committee (2023AW048).

### 2.2 Animal grouping

The animal experiment was described in detail in our previously published article (23). Briefly, a total of 20 21-day-old lactating female C57BL/6 mice were randomly assigned to four groups (*n* = 5 per group): the normal control group (CON), the EGCG intervention group on a normal diet (EGCG), the HFD group, and the HFD with the EGCG intervention group (HFDEGCG). Animals were randomly assigned to four experimental groups using a computer-generated randomization list to minimize selection bias. The normal control group received a standard diet and regular drinking water, while the HFD group was provided with an HFD and regular drinking water. Based on the results of our previous study (19), the intervention protocol for the EGCG group involved supplementing the drinking water with 2 mg/mL EGCG. Based on the average water consumption and body weight of the mice, the effective dose of EGCG consumed was calculated to be approximately 267 mg/kg of body weight per day. The EGCG intervention group was divided into two subgroups: one that received a standard diet and the other that received an HFD. Throughout the study, the drinking water and feed for the EGCG intervention group were refreshed daily. In contrast, the control and HFD groups received regular drinking water and feed, which were replaced every 2 days. Additionally, body weight and vaginal opening condition of the mice in each group were monitored. To minimize observer bias, blinding was used during the measurement of key outcomes, such as vaginal opening. The investigators performing the measurements were unaware of the group assignments during data collection, which further reduced potential bias in the results. Mice were anesthetized by intraperitoneal injection of sodium pentobarbital (50 mg/kg). Following anesthesia, euthanasia was performed by cervical dislocation. Blood samples were collected from the orbital region for serum metabolomics analysis when the mice reached 40 days of age.

### 2.3 UHPLC–MS/MS analysis

Serum samples (100 μL) were protein-precipitated with cold organic solvent (acetonitrile: methanol, 1:1 v/v). After vortexing for 30 s and ultrasonication at 5 °C and 40 kHz for 30 min, samples were incubated at −20 °C for 30 min and centrifuged at 13,000 *g* and 4 °C for 15 min. Supernatants were dried under nitrogen gas and reconstituted in acetonitrile:water (1:1 v/v,100 μL), ultrasonicated at 5 °C for 5 min, and recentrifuged at 13,000 g and 4 °C for 5 min. The final supernatants were transferred to vials for analysis. A pooled quality control (QC) sample was generated from equal-volume aliquots from all individual samples. QC injections were performed after every 10 analytical runs to monitor system stability (25). Chromatographic conditions and mass spectrometry parameters are described in the Supplementary materials.

### 2.4 Data preprocessing and database search

For data preprocessing, the metabolomics analysis software Progenesis QI was employed, incorporating crucial procedures such as baseline correction, peak identification and integration, retention time calibration, and peak alignment. This sequence of steps led to the creation of a data matrix that included retention time, mass-to-charge ratio, and peak intensity. A mixture of internal standards (L-2-chlorophenylalanine for positive ion mode; heptadecanoic acid for negative ion mode) was added to each sample prior to extraction to monitor procedural performance. The resultant mass spectrometry data were subsequently matched against public databases such as HMDB^1^ (26) and Metlin^2^ (27), as well as a proprietary database created by MajorBio, to ensure precise metabolite annotation. To address systematic errors typical of the experimental setup, total sum normalization was applied to standardize the mass spectrometry signal intensities. Metabolic features with a relative standard deviation > 30% in the quality control samples were removed to ensure data quality. The remaining high-quality data were log10-transformed and mean-centered for subsequent multivariate statistical analysis. These data were uploaded to the MajorBio cloud platform^3^ for extensive further analysis (28).

### 2.5 Analysis of differential metabolites

The data matrix, after preprocessing and variance filtering, was utilized for the initial screening phase. Multivariate statistical modeling was conducted using the R package “ropls” (version 1.6.2). Principal component analysis (PCA), an unsupervised method, was first performed to visualize general clustering, trends, and outliers within the metabolomic dataset. Subsequently, partial least squares-discriminant analysis (PLS-DA), a supervised method, was applied to maximize the separation between pre-defined groups and to identify metabolites that contributed significantly to the group discrimination. The robustness and predictive ability of the PLS-DA model were evaluated through a seven-fold cross-validation procedure, which iteratively partitions the data into training and validation sets to prevent model overfitting and to provide unbiased performance estimates. The permutation test is a validation method that assesses the risk of the PLS-DA model being overfit. In this test, the group labels (e.g., HFD or HFDEGCG) of the samples are randomly shuffled hundreds of times, and new models are built each time. The resulting R^2^Y and Q^2^ values from these random models form a distribution. A robust and valid original model is indicated when its Q^2^ value (far right on the x-axis) is significantly higher than all the Q^2^ values from the permuted models and when the regression line of the permuted Q^2^ values intersects the y-axis below zero. This finding confirms that the group separation we observed is statistically meaningful and not a result of random finding. Furthermore, Student’s *t*-test and fold change calculations were performed. Differential metabolites were identified based on the variable importance for the projection (VIP) values from the PLS-DA model and *p*-values from the *t*-test, with metabolites exhibiting VIP > 1 and *p* < 0.05 being classified as differential metabolites (29). The pathway enrichment analysis and pathway topological analysis were employed to identify significantly enriched metabolic pathways, utilizing the Kyoto Encyclopedia of Genes and Genomes (KEGG) database (30, 31). To identify potential protein targets for molecular docking, we focused on enzymes directly involved in the metabolic pathways of the significantly differential metabolites. Specifically, the differential metabolites were mapped to the KEGG database to identify their associated biosynthetic and degradation pathways. The enzymes that catalyze the immediate biochemical reactions producing (upstream) or consuming (downstream) these metabolites were extracted.

### 2.6 Molecular docking

The upstream and downstream proteins associated with the differential metabolites were identified through a comprehensive search in the AlphaFold V2.0 and RCSB databases, as well as predictions using AlphaFold V3.0 (32, 33). The target protein underwent preprocessing on the AutoDock platform, which included the removal of water molecules and the addition of hydrogen atoms, resulting in the generation of a receptor file in PDBQT format (34). The three-dimensional structure of the ligand molecule, EGCG, was retrieved from the PubChem database^4^ (35). Utilizing the OpenBabelGUI software, the initial SDF file was converted into PDB format. Then, the EGCG molecule underwent similar preprocessing steps, including the removal of water molecules and the addition of hydrogen atoms, before being exported as a ligand file in PDBQT format. Subsequently, the prepared receptor and ligand files were imported into the AutoDock platform for parameter configuration. The docking grid was centered on the target protein, ensuring comprehensive coverage of the protein within the docking box. Molecular docking simulations between EGCG and the proteins related to metabolite pathways were performed using AutoDock Vina to calculate the minimum binding energy, thereby elucidating the interaction mechanisms and potential regulatory functions. The results were visualized using PyMol 2.5 software (36). Based on the docking outcomes, key targets exhibiting strong binding abilities with the differential metabolites were selected, and their biological relevance was further corroborated through literature review and database analyses.

## 3 Results

### 3.1 PLS-DA analysis

**Figure 1:**
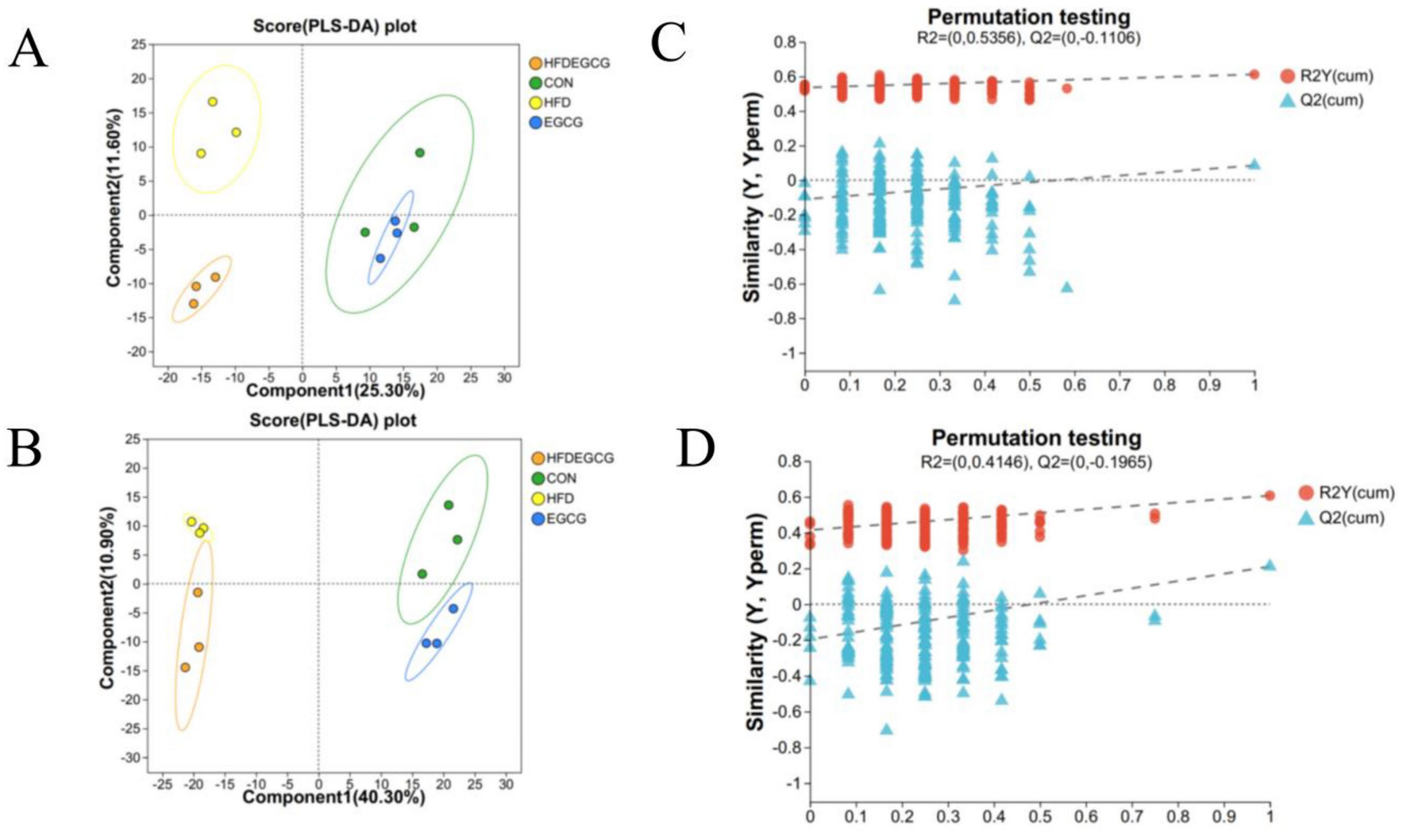
PLS-DA analysis and permutation testing reveal group separations and model validity in metabolomic profiles. **(A,B)** Partial least squares-discriminant analysis (PLS-DA) score plots. Plots show the separation of serum metabolic profiles among the four experimental groups in both positive **(A)** and negative **(B)** ionization modes. Each point represents an individual mouse sample. The ellipses represent the 95% confidence intervals for each group. Groups are CON (normal diet, green circles), HFD (high-fat diet, yellow circles), EGCG (normal diet plus EGCG, blue circles), and HFDEGCG (high-fat diet plus EGCG, orange circles). Component 1 and Component 2 are latent variables that best separate the groups, and the percentage values indicate the proportion of total variance explained by each component. **(C,D)** Permutation test plots (200 permutations) for the PLS-DA models in positive **(C)** and negative **(D)** mode. The plots validate that the original models (far right) are robust and not overfitted. The y-axis represents the values of the goodness-of-fit (R^2^Y, blue triangles) and the goodness-of-prediction (Q^2^, red dots) parameters. The dashed lines represent the regression lines for the permuted R^2^Y and Q^2^ values. The fact that the original Q^2^ value (far right) is significantly higher than all permuted Q^2^ values and that the regression line of the permuted Q^2^ values has a negative intercept confirms the statistical validity and predictive power of the model.

Our previous animal study demonstrated that EGCG can prevent obesity-induced precocious puberty (23). In the present study, PLS-DA was employed to characterize the serum metabolite profiles of mice in the four groups. The PLS-DA score plots, depicted in both positive mode (Figure 1A) and negative mode (Figure 1B), revealed marked differences among the four groups, suggesting substantial intergroup differences in their serum metabolic profiles. In the positive mode, components 1 and 2 of the PLS-DA model explained 25.3 and 11.6% of the total variance, respectively. Conversely, in the negative mode, these components accounted for 40.3 and 10.9% of the variance, respectively. The robustness and validity of the model were assessed through permutation testing, which yielded R^2^ = 0.5356 and Q^2^ = −0.1106 in positive mode (Figure 1C) and R^2^ = 0.4146 and Q^2^ = −0.1965 in negative mode (Figure 1D). These results indicated that the metabolic differences between the HFDEGCG group and the HFD group were statistically significant rather than an overfitting result of the model, revealing that the EGCG intervention significantly altered the serum metabolite profile during the development of obesity-induced precocious puberty in mice.

### 3.2 Screening of differential metabolites

**Figure 2:**
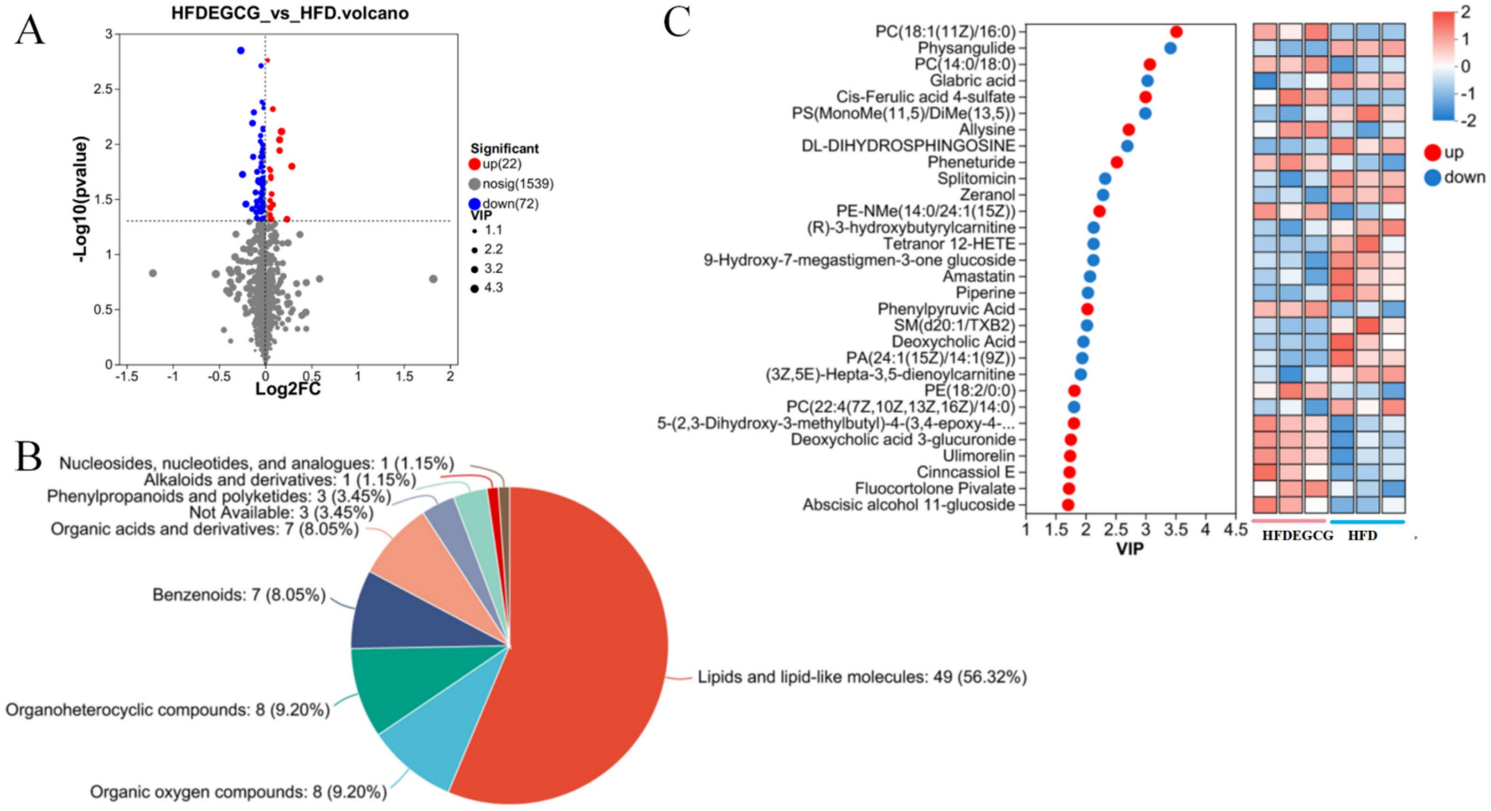
Analysis of differential metabolites between the HFDEGCG and HFD groups. **(A)** Volcano plot displaying the metabolic changes. The *x*-axis represents the log2 (Fold Change) between the HFDEGCG and HFD groups, and the *y*-axis represents the −log10 of the *p*-value. Significantly upregulated metabolites (red dots, *n* = 22) are defined as having a variable importance in projection (VIP) score of > 1.0 and a *p*-value of < 0.05. Significantly downregulated metabolites (blue dots, *n* = 72) meet the same criteria in the negative direction. Grey dots represent metabolites with no significant difference (*n* = 1,539). **(B)** Pie chart showing the classification of the 94 significant differential metabolites based on their superclass. Lipids and lipid-like molecules (56.32%) constituted the most abundant category. **(C)** Left panel: VIP scores from the PLS-DA model for the top 30 metabolites driving group separation. Red dots represent metabolites upregulated in the HFDEGCG group, and blue dots represent downregulated metabolites. Right panel: Heatmap of the relative abundance of these top 30 metabolites across individual samples in the HFDEGCG and HFD groups. A red color indicates a higher relative abundance, and a blue color indicates a lower relative abundance.

The analysis of the differences in serum metabolites between the HFDEGCG group and the HFD group revealed significant differences in 94 metabolites between the two groups, of which 22 metabolites were upregulated and 72 were downregulated (Figure 2A). These differentially expressed metabolites were classified, and among them, lipid and lipid-like molecules accounted for 56.32%, making it the most abundant category (Figure 2B).

According to the screening criteria (VIP > 1, *p* < 0.05), the top 30 differential metabolites were selected. Compared with the HFD group, the HFDEGCG group showed upregulation of 14 differential metabolites, including PC(18:1(11Z)/16:0), PC(14:0/18:0), cis-ferulic acid 4-sulfate, allysine, pheneturide, PE-NMe(14:0/24:1(15Z)), phenylpyruvic acid, PE(18:2/0:0), 5-(2,3-dihydroxy-3-methylbutyl)-4-(3,4-epoxy-4-methylpentanoyl)-3,4-dihydroxy-2-isopentanoyl-2-cyclopenten-1-one, ulimorelin, Cinncassiol E, fluocortolone pivalate, and abscisic alcohol 11-glucoside. There were 16 downregulated differential metabolites, including physangulide, glabric acid, PS(MonoMe(11,5)/DiMe(13,5)), DL-dihydrosphingosine, splitomicin, zeranol, (R)-3-hydroxybutyrylcarnitine, tetranor 12-HETE, 9-hydroxy-7-megastigmen-3-one glucoside, amastatin, piperine, SM(d20:1/TXB2), deoxycholic acid, PA(24:1(15Z)/14:1(9Z)), (3Z,5E)-hepta-3,5-dienoylcarnitine, and PC(22:4(7Z,10Z,13Z,16Z)/14:0) (Table 1; Figure 2C).

**Table 1:** Differential metabolites between the HFDEGCG and HFD groups.

Changes in the expression levels of differential metabolites	Differential metabolites	VIP	*p* value
Upregulated metabolites	PC(18:1(11Z)/16:0)	3.515	0.008
	PC(14:0/18:0)	3.013	0.009
	Cis-Ferulic acid 4-sulfate	2.966	0.016
	Allysine	2.700	0.048
	Pheneturide	2.508	0.011
	PE-NMe(14:0/24:1(15Z))	2.177	0.036
	Phenylpyruvic Acid	2.032	0.005
	PE(18:2/0:0)	1.811	0.029
	5-(2,3-Dihydroxy-3-methylbutyl)-4-(3,4-epoxy-4-methylpentanoyl)-3,4-dihydroxy-2-isopentanoyl-2-cyclopenten-1-one	1.748	0.020
	Deoxycholic acid 3-glucuronide	1.709	0.017
	Ulimorelin	1.689	0.021
	Cinncassiol E	1.676	0.038
	Fluocortolone Pivalate	1.751	0.048
	Abscisic alcohol 11-glucoside	1.640	0.044
Downregulated metabolites	Physangulide	3.388	0.001
	Glabric acid	2.968	0.035
	PS(MonoMe(11,5)/DiMe(13,5))	2.917	0.019
	DL- dihydrosphingosine	2.640	0.006
	Splitomicin	2.269	0.013
	Zeranol	2.295	0.005
	(R)-3-hydroxybutyrylcarnitine	2.115	0.028
	Tetranor 12-HETE	2.071	0.039
	9-Hydroxy-7-megastigmen-3-one glucoside	2.101	0.018
	Amastatin	2.038	0.047
	Piperine	1.962	0.022
	SM(d20:1/TXB2)	1.993	0.042
	Deoxycholic Acid	1.899	0.037
	PA(24:1(15Z)/14:1(9Z))	1.902	0.021
	(3Z,5E)-Hepta-3,5-dienoylcarnitine	1.892	0.033
	PC(22:4(7Z,10Z,13Z,16Z)/14:0)	1.832	0.040

### 3.3 Differential metabolites analyzed through KEGG pathway enrichment

**Figure 3:**
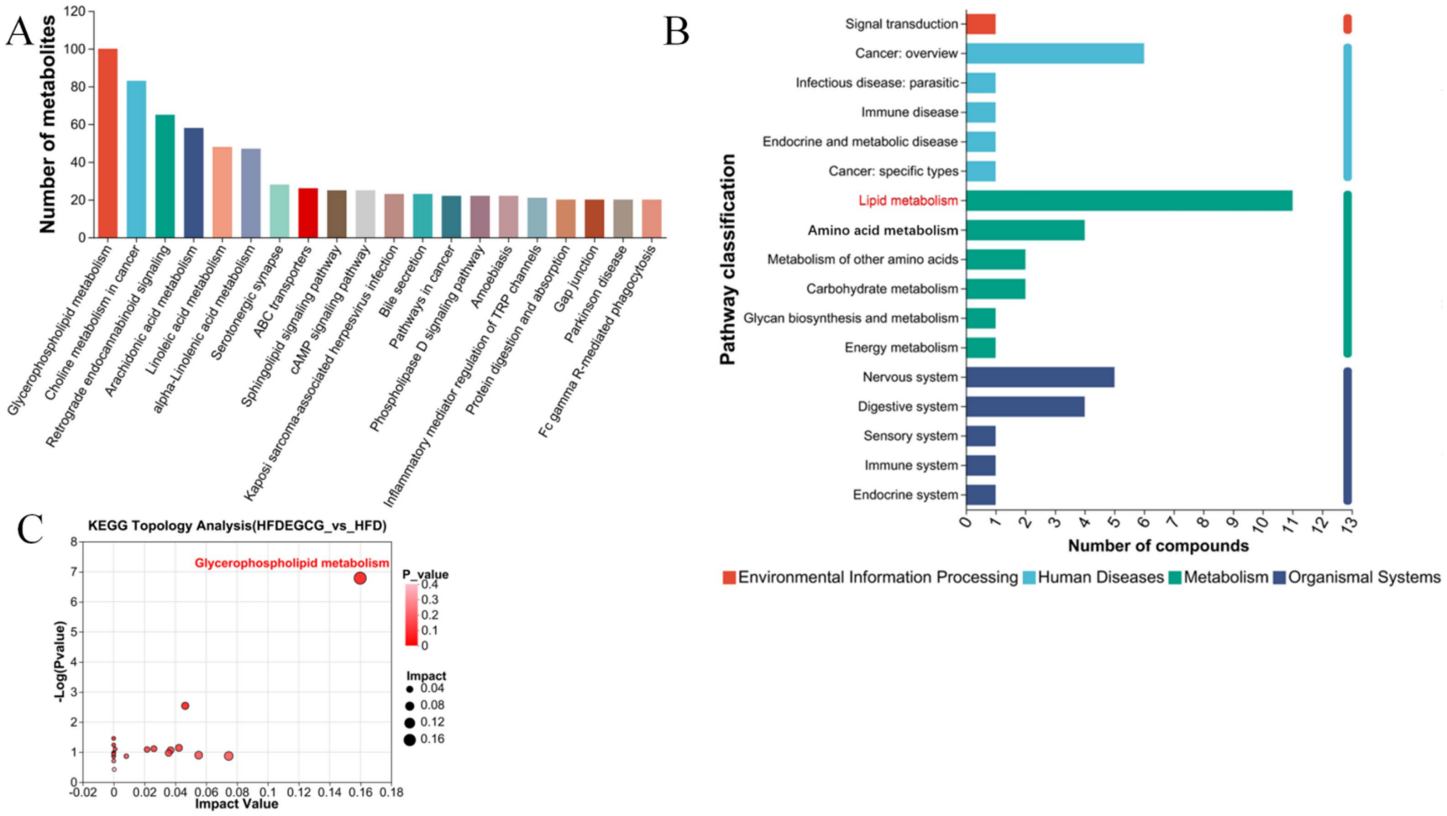
KEGG pathway enrichment and topology analysis of differential metabolites between HFDEGCG and HFD groups. **(A)** Bar plot of Kyoto Encyclopedia of Genes and Genomes (KEGG) pathway enrichment. The top 20 significantly enriched pathways are shown. The *X*-axis shows the pathway names. The *Y*-axis represents the Rich Factor (the number of differential metabolites mapped to the pathway divided by the total number of metabolites detected in that pathway). A larger Rich Factor indicates a greater degree of enrichment. **(B)** Bar chart of pathway categorization. The *X*-axis represents the number of compounds. The *Y*-axis shows the broader categories of the enriched KEGG pathways. **(C)** Scatter plot of pathway impact from topology analysis. The *X*-axis (Pathway Impact) calculates the cumulative importance of the differential metabolites within a pathway, based on their centrality in the metabolic network. The *Y*-axis represents the −log10 of the *p*-value from the enrichment analysis. Each point represents a metabolic pathway. Glycerophospholipid metabolism (highlighted in red) was identified as the most significantly altered pathway, possessing both high statistical significance (low *p*-value) and a high topological impact within the network. This identifies it as the key pathway mediating EGCG’s effect.

The KEGG pathway enrichment analysis of the differential metabolites identified the top 20 significantly enriched pathways based on their *p*-value, which included glycerophospholipid metabolism, choline metabolism in cancer, retrograde endocannabinoid signaling, arachidonic acid metabolism, linoleic acid metabolism, and alpha-linolenic acid metabolism. (Figure 3A). To further understand the biological functions involved, the enriched pathways were categorized into broader KEGG functional classes, as shown in the pathway classification plot (Figure 3B). This analysis revealed that lipid metabolism was a key category affected by EGCG. The KEGG topological analysis further suggested that glycerophospholipid metabolism was a key metabolic pathway associated with EGCG’s preventive effect on obesity-induced precocious puberty (Figure 3C).

Comparison of the glycerophospholipid differential metabolites between the HFDEGCG and HFD groups revealed significant differences in the serum concentrations of eight glycerophospholipid metabolites. The EGCG intervention markedly influenced the concentration of several glycerophospholipid metabolites, including phosphatidic acid (PA), phosphatidylcholine (PC), lysophosphatidylcholine (LysoPC), phosphatidylserine (PS), and phosphatidyl ethanolamine (PE). Specifically, EGCG treatment elevated the levels of PC(18:1(11Z)/16:0), PC(14:0/18:0), and PE-NMe(14:0/24:1(15Z)) in mice on the high-fat diet. Meanwhile, it significantly decreased the levels of PA(24:1(15Z)/14:1(9Z)), PC(22:4(7Z, 10Z, 13Z, 16Z)/14:0), LysoPC(20:5(5Z, 8Z, 11Z, 14Z, 17Z)/0:0), PS(20:4(8Z, 11Z, 14Z, 17Z)/14:1(9Z)), and LysoPC(15:0/0:0) in mice on the high-fat diet. (Table 2). These results suggested that the preventive effect of EGCG may be associated with the regulation of these eight glycerophospholipid metabolites.

**Table 2:** Differential metabolites of glycerophospholipids between the HFDEGCG and HFD groups.

Changes in the expression levels of glycerophospholipid differential metabolites	Differential metabolites of glycerophospholipids	VIP	*p* value
Upregulated metabolites	PC(18:1(11Z)/16:0)	3.515	0.008
	PC(14:0/18:0)	3.013	0.009
	PE-NMe(14:0/24:1(15Z))	2.177	0.036
Downregulated metabolites	PA(24:1(15Z)/14:1(9Z))	1.902	0.021
	PC(22:4(7Z, 10Z, 13Z, 16Z)/14:0)	1.832	0.040
	LysoPC(20:5(5Z, 8Z, 11Z, 14Z, 17Z)/0:0)	1.454	0.041
	PS(20:4(8Z, 11Z, 14Z, 17Z)/14:1(9Z))	1.385	0.048
	LysoPC(15:0/0:0)	1.095	0.023

### 3.4 Molecular docking of EGCG and differential metabolite-related proteins

Molecular docking analysis was conducted to assess the potential interaction between EGCG and key upstream catalytic enzymes or downstream regulatory targets that directly govern the levels of identified glycerophospholipid metabolites (Supplementary Figure S2). These key proteins included phosphatidylserine decarboxylase (PISD), phospholipase D (PLD), phosphatidylserine synthase (PTDSS), phosphatidyl-N-methylethanolamine N-methyltransferase, phospholipase A2, phosphatidylcholine--sterol O-acyltransferase, phospholipase C, and CDP-diacylglycerol synthase. Binding affinity was evaluated based on docking binding energy, where more negative values indicate higher affinity. A binding energy threshold of < −5.0 kcal/mol is generally considered indicative of good binding activity (37). The results demonstrated strong binding potential between EGCG and all eight proteins analyzed (Table 3). This finding provided preliminary validation of the metabolomics results and further supported the proposition that PISD, PLD, and PTDSS may serve as key targets through which EGCG prevents obesity-induced precocious puberty.

**Table 3:** Molecular docking binding energy between EGCG and target proteins.

Protein name	Binding energy (kcal/mol)
Phosphatidylserine decarboxylase	−9.7
Phospholipase D	−9.3
Phosphatidylserine synthase	−9.3
Phosphatidyl-N-methylethanolamine N-methyltransferase	−8.5
Phospholipase A2	−8.2
Phosphatidylcholine--sterol O-acyltransferase	−8.2
Phospholipase C	−8.1
CDP-diacylglycerol synthase	−7.4

**Figure 4:**
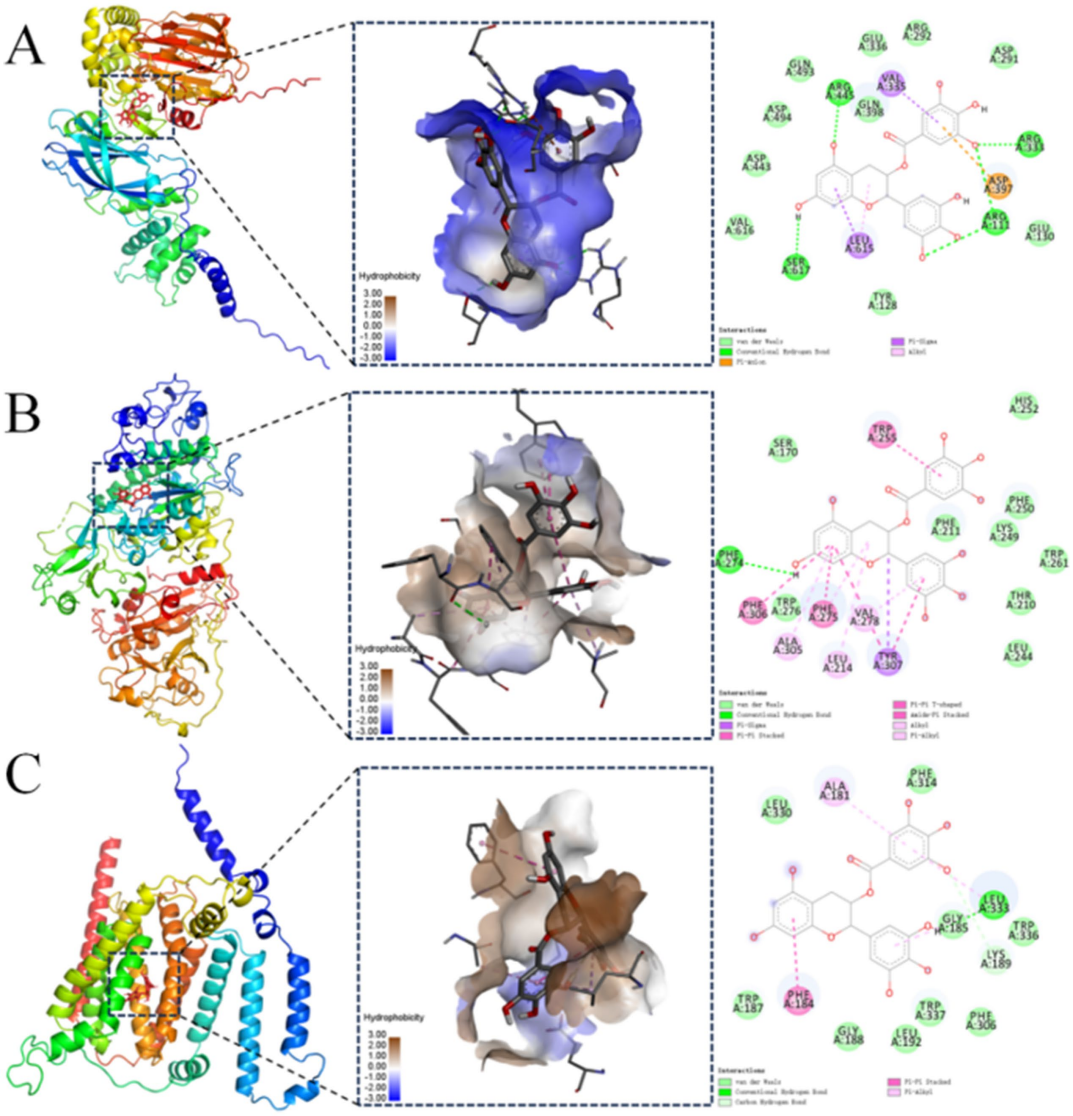
Molecular docking visualization between the protein and the EGCG ligand. Visualizations of molecular docking between EGCG and: **(A)** phosphatidylserine decarboxylase (PISD); **(B)** phospholipase D (PLD); **(C)** phosphatidylserine synthase (PTDSS).

Molecular docking results for proteins exhibiting binding energies below −9.0 kcal/mol with EGCG were visualized in Figure 4. In the figure, brown regions surrounding the ligand represented hydrophobic protein residues, while blue regions denoted hydrophilic residues. These results suggested that PISD, PLD, and PTDSS each formed strong interactions with EGCG.

The molecular docking analysis indicated a binding energy of −9.7 kcal/mol for the PISD–EGCG complex, which was primarily stabilized through hydrogen bonding, hydrophobic interactions, and electrostatic forces. The lowest-energy conformation revealed EGCG bound predominantly within a hydrophilic pocket of PISD. Specific interactions included hydrogen bonds between EGCG and PISD residues Arg445, Arg333, Ser617, and Arg111. Hydrophobic interactions involved an alkyl contact with Leu615 and*π*-sigma interactions with Val335 and Leu615. Additionally, Asp397 formed aπ-anion electrostatic interaction with EGCG (Figure 4A). These interactions collectively conferred strong binding affinity.

The molecular docking binding energy of PLD and EGCG was −9.3 kcal/mol. The binding was mediated mainly by hydrogen bonds and hydrophobic interactions. In the lowest-energy conformation, EGCG occupied a hydrophobic pocket on PLD. A hydrogen bond formed between Phe274 of PLD and EGCG. Hydrophobic interactions included *π*-alkyl contacts with Ala305, Leu214, Phe275, and Val278, and π-π stacking interactions with Phe306, Phe275, Trp255, and Tyr307. Tyr307 also engaged in a π-sigma interaction (Figure 4B). These forces enabled strong EGCG-PLD binding.

The molecular docking binding energy of PTDSS and EGCG was −9.3 kcal/mol. The binding mainly involved hydrogen bonds and hydrophobic interactions. The conformation with the lowest docking binding energy showed that most of EGCG was bound to the hydrophobic pocket of the PTDSS protein. Leu333 on PTDSS formed a hydrogen bond with EGCG, and Lys189 formed an unconventional hydrogen bond with PTDSS, Ala181 and Leu33 formed π-alkyl hydrophobic bond forces with EGCG, and Phe184 formed a π-π stacking hydrophobic bond force with EGCG. These interactions facilitated strong EGCG-PTDSS binding (Figure 4C). These findings provided preliminary computational support for the metabolomics results and suggested that PISD, PLD, and PTDSS may serve as potential key targets worthy of further experimental validation.

### 3.5 The impact of EGCG on lipid metabolism and its potential link to the prevention of obesity-induced precocious puberty

**Figure 5:**
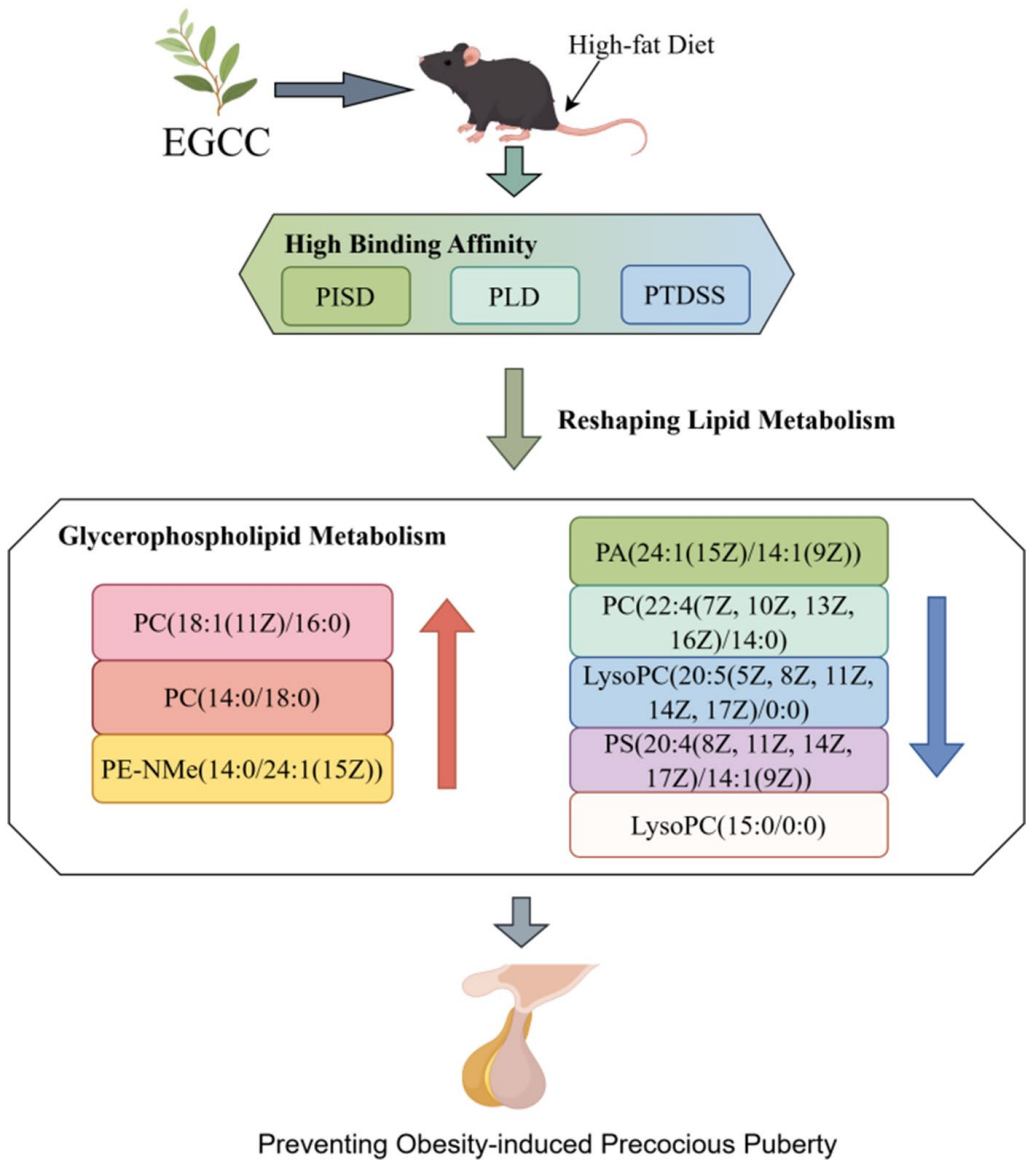
The impact of EGCG on lipid metabolism and its potential link to the prevention of obesity-induced precocious puberty. This figure illustrates the altered glycerophospholipid metabolites following EGCG intervention in a mouse model of obesity-induced precocious puberty. The molecular docking results suggest a potential mechanism, showing that EGCG exhibits a high binding affinity to key lipid-metabolizing enzymes (PISD, PLD, and PTDSS). The metabolomic analysis revealed that EGCG intervention was associated with an elevation in the levels of glycerophospholipids such as PC(18:1(11Z)/16:0), PC(14:0/18:0), and PE-NMe(14:0/24:1(15Z)) and a reduction in the levels of lipid metabolites including PA(24:1(15Z)/14:1(9Z)), PC(22:4(7Z, 10Z, 13Z, 16Z)/14:0), LysoPC(20:5(5Z, 8Z, 11Z, 14Z, 17Z)/0:0), PS(20:4(8Z, 11Z, 14Z, 17Z)/14:1(9Z)), and LysoPC(15:0/0:0). These findings suggest that the modulation of lipid metabolism may be associated with the preventive effect of EGCG.

Figure 5 illustrates the altered glycerophospholipid metabolites following EGCG intervention in a mouse model of obesity-induced precocious puberty. The molecular docking results suggest a potential mechanism, showing that EGCG exhibits a high-binding affinity to key lipid-metabolizing enzymes (PISD, PLD, and PTDSS). The metabolomic analysis revealed that EGCG intervention was associated with an elevation in the levels of glycerophospholipids, such as PC(18:1(11Z)/16:0), PC(14:0/18:0), and PE-NMe(14:0/24:1(15Z)), and a reduction in the levels of lipid metabolites, including PA(24:1(15Z)/14:1(9Z)), PC(22:4(7Z, 10Z, 13Z, 16Z)/14:0), LysoPC(20:5(5Z, 8Z, 11Z, 14Z, 17Z)/0:0), PS(20:4(8Z, 11Z, 14Z, 17Z)/14:1(9Z)), and LysoPC(15:0/0:0). Taken together, these findings suggested that EGCG exerts its preventive effect on obesity-induced precocious puberty via high-affinity binding to key lipid-metabolizing enzymes (PISD, PLD, and PTDSS) and subsequent regulation of glycerophospholipid homeostasis.

## 4 Discussion

Precocious puberty is the second most prevalent pediatric endocrine disorder following childhood obesity, and it significantly impacts children’s growth as well as their physical and mental health in adulthood (2). While our previous study demonstrated the effect of EGCG in preventing obesity-induced precocious puberty (23), the potential mechanisms behind remained unclear. The present study aims to elucidate the underlying molecular mechanisms based on serum metabolomics and molecular docking. Small molecule metabolites abundant in the serum can offer comprehensive insights into disease onset and drug effects (38, 39). In the present study, UHPLC–MS/MS combined with multivariate statistical analysis was employed to investigate alterations in serum metabolites induced by EGCG. Our results demonstrated that the EGCG intervention during the HFD feeding period significantly altered the serum metabolite profile, suggesting a metabolic basis for its preventive effect. A total of 94 metabolites were significantly altered, among which lipids and lipid-like molecules constituted the most abundant category (56.32%).

The KEGG pathway enrichment analysis revealed that lipid metabolism, particularly glycerophospholipid metabolism, was the key pathway affected by EGCG. Furthermore, KEGG topological analysis suggested that glycerophospholipid metabolism was the key metabolic pathway underlying EGCG’s preventive effect on obesity-induced precocious puberty, reflecting its role in regulating lipid homeostasis.

**Figure 6:**
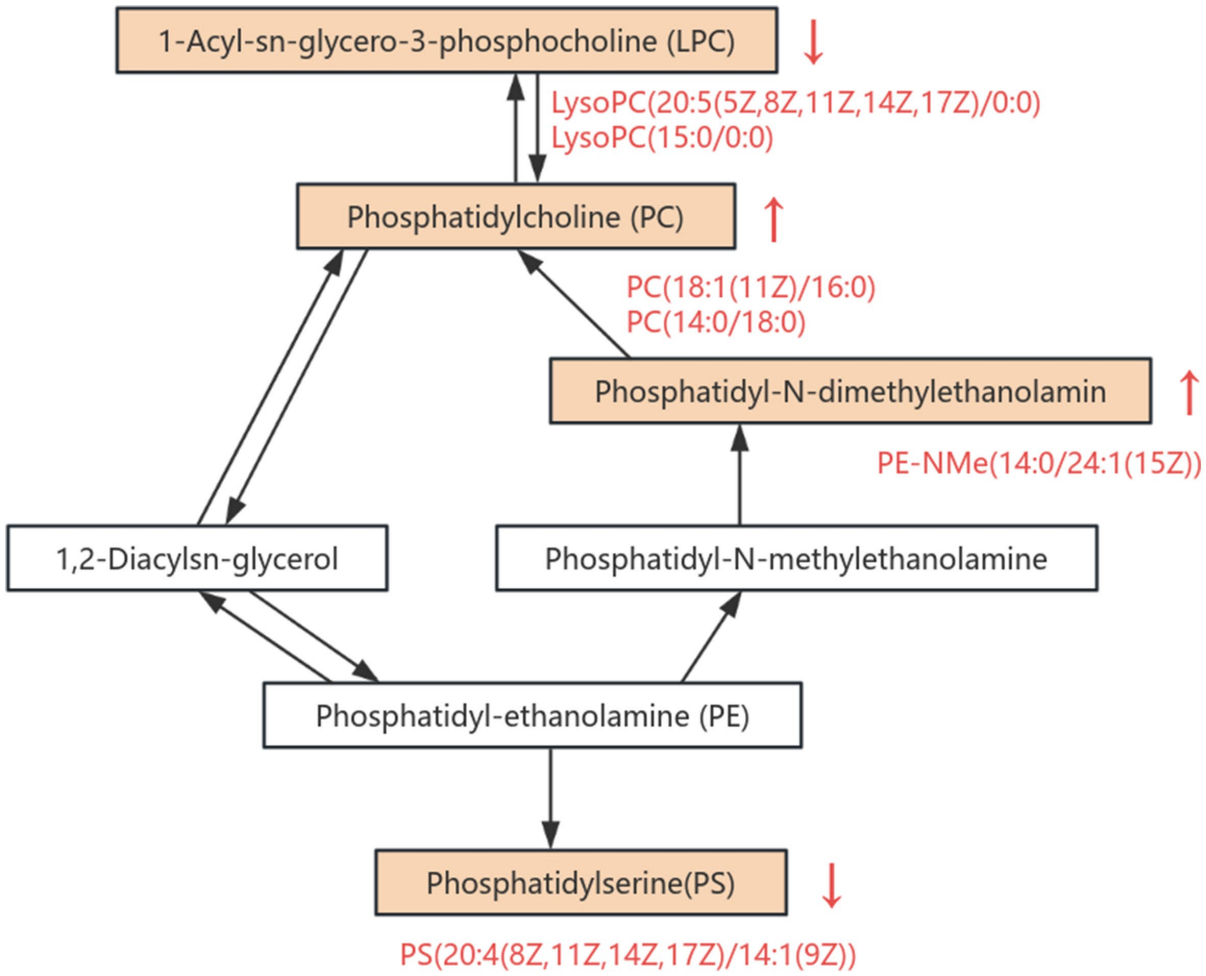
Overview of altered metabolites in the glycerophospholipid metabolic pathway. This pathway diagram illustrates the differential regulation of key metabolites involved in glycerophospholipid metabolism between HFDEGCG and HFD groups. Significantly upregulated metabolites are indicated in red text with upward arrows (↑), while downregulated metabolites are labeled with downward arrows (↓).

Lipids are the most diverse class of small-molecule compounds in eukaryotes. They not only perform structural functions but also participate in processes such as cell transport, energy storage, and signal transduction (40). The study found that, compared with the control group, the levels of glycerophospholipid metabolites PC(18:1(11Z)/16:0), PC (14:0/18:0), and PE-NMe(14:0/24:1(15Z)) in mice significantly increased after EGCG intervention, while the levels of PA(24:1(15Z)/14:1(9Z)), PC(22:4(7Z, 10Z, 13Z, 16Z)/14:0), LysoPC(20:5(5Z, 8Z, 11Z, 14Z, 17Z)/0:0), PS(20:4(8Z, 11Z, 14Z, 17Z)/14:1(9Z)), and LysoPC(15:0/0:0) significantly decreased (Figure 6). By modulating the levels of key glycerophospholipids such as PA, PC, LysoPC, PS, and PE, EGCG influences membrane composition and permeability, thereby affecting cellular physiological functions. As the main lipid components of cell membranes, glycerophospholipids serve as important biomarkers for evaluating lipid metabolism disorders (41). Research has demonstrated that glycerophospholipids play a crucial role in conditions such as arteriosclerosis, diabetes, cancer, inflammation, and dyslipidemia (42, 43).

Recently, a seminal study by Elvira et al. provided direct evidence linking hypothalamic lipid metabolism to the timing of puberty (44). Using a female rat model, they demonstrated that overweight conditions linked to precocious puberty are associated with significant changes in the hypothalamic profiles of specific lipid species, including fatty-acyls, bile acid derivatives, and several glycerophospholipids, during the juvenile-pubertal transition. Our findings, which reveal a profound reshaping of serum glycerophospholipid metabolism by EGCG, exhibit a remarkable congruence with their central nervous system findings. This convergence across different biological compartments (serum vs. hypothalamus) and species (mouse vs. rat) strongly suggests that the dysregulation of lipid metabolism is a fundamental pathological feature of obesity-induced precocious puberty. While our study measured metabolites in the periphery, it is plausible that the significant changes we observed (e.g., in PC, PA, and LysoPC species) reflect systemic metabolic shifts that are closely related to, or even directly influence, the lipid milieu within the hypothalamus. LysoPCs, for instance, are known signaling molecules that can affect inflammatory processes and potentially cross the blood–brain barrier (45). The findings from Elvira et al. thereby provide a crucial missing link, offering a more tangible connection between our serum metabolome results and the central mechanism controlling puberty onset. They lead us to hypothesize that EGCG’s preventive effect may be mediated not only by its systemic anti-obesity action but also by its capacity to normalize a disrupted lipid metabolism, which is evident both peripherally and centrally, ultimately contributing to the appropriate timing of the HPGA activation.

Through serum metabolomics analysis, potential protein targets for EGCG in preventing obesity-induced precocious puberty—namely, PISD, PLD, and PTDSS—were identified. Molecular docking was subsequently employed for preliminary verification, selecting proteins with high-binding affinity based on a binding free energy threshold of less than −9.0 kcal/mol. The *de novo* synthesis pathway of phosphatidylcholine and phosphatidyl ethanolamine is the Kennedy pathway (46), which involves three steps. First, the choline or ethanolamine is phosphorylated to form phosphocholine and phosphoethanolamine. Second, the products are converted into CDP-choline and CDP-ethanolamine. The third step involves the combination of the products from the second step with diacylglycerol, resulting in the formation of phosphatidylcholine and phosphatidyl ethanolamine (47). In parallel, an alternative and distinct pathway exists for phosphatidyl ethanolamine synthesis. PISD can catalyze the decarboxylation of serine in phospholipids to produce phosphatidyl ethanolamine (48). Girisha et al. found that PISD plays a crucial role in human development (49); mutations in the PISD gene can lead to dysplasia of the vertebral epiphysis. Moreover, PISD is essential for maintaining the mitochondria integrity and muscle mass in skeletal muscle, playing a significant role in maintaining phospholipid homeostasis in adult skeletal muscle (50). PLD can catalyze the hydrolysis of phosphatidylcholine to generate phosphatidic acid and choline. Phosphatidic acid is an important metabolite in the PLD signaling pathway and serves as an active substrate for various phosphatidylinositol 3-kinase (PI3K)/protein kinase B (Akt) signaling pathways in organisms. Previous basic research has indicated that the PI3K/Akt signaling pathway plays a vital role in the pathogenesis of precocious puberty, making it a potential therapeutic target (24, 51). Furthermore, phosphatidylserine is synthesized from phosphatidylcholine or phosphatidyl ethanolamine through an exchange reaction with serine, and the catalytic enzyme for this reaction is PTDSS (52, 53). PTDSS plays a vital role in metabolic diseases. In adipose tissue, the expression of PTDSS 2 is positively correlated with body mass index and waist-to-hip ratio (54). Nevertheless, the significance of this enzyme in obesity has not yet been fully clarified. Some studies suggest that PTDSS 2 and phosphatidylserine may be associated with obesity by influencing thermogenesis or other mechanisms (55). In conclusion, EGCG may restore lipid metabolic homeostasis by targeting key nodes—PISD, PLD, and PTDSS—in glycerophospholipid metabolism, thereby ameliorating lipid disorders and contributing to the prevention of obesity-induced precocious puberty.

The results of the present study provide molecular-level theoretical support for the use of EGCG as a natural bioactive intervention strategy. Meanwhile, they suggest that phospholipid metabolism may be an important bridge connecting obesity and precocious puberty, providing novel directions for nutritional research aimed at utilizing dietary bioactive components to prevent this condition. Recent lipidomic analyses have identified specific lipid species, such as ceramides and phosphoinositols, as biomarkers for central precocious puberty and metabolic disturbances in adolescents (56, 57). These findings align with our observation that EGCG modulates lipid metabolism pathways, particularly glycerophospholipid metabolism, to mitigate obesity-induced precocious puberty. Additionally, studies have demonstrated that lipid-sensing pathways in the central nervous system play a crucial role in pubertal timing (12, 44). The modulation of these pathways by dietary compounds like EGCG could offer novel therapeutic strategies for managing early puberty onset.

Beyond glycerophospholipid metabolism, our pathway analysis also highlighted the potential involvement of other lipid-related pathways, including arachidonic acid metabolism and sphingolipid signaling. Although these pathways did not emerge as the top-enriched pathways in our model, their biological significance in puberty and metabolism warrants discussion. Arachidonic acid is a precursor for eicosanoids, which are potent signaling molecules involved in inflammation and steroidogenesis (58). Dysregulation of arachidonic acid metabolism has been linked to metabolic disorders and could indirectly influence the HPGA through inflammatory processes (11). Similarly, sphingolipids, such as ceramides and sphingomyelins, are not only structural components but also key regulators of insulin resistance, apoptosis, and stress responses (59, 60). Recent lipidomics studies revealed ceramide biomarkers for detecting central precocious puberty in girls (57). While our study primarily focuses on the glycerophospholipid pathway due to the strength of the metabolic signature and docking results, the alteration of these parallel pathways suggests a complex, interconnected lipid network that is disrupted in obesity-induced precocious puberty and modulated by EGCG. The interplay between glycerophospholipid, arachidonic acid, and sphingolipid metabolism represents an interesting area for future research to fully elucidate the lipid-centric mechanisms controlling pubertal timing. It is important to note that the significant anti-obesity effect of EGCG, as reported in our previous study (23), likely plays a primary role in delaying puberty onset. The specific serum metabolome alterations observed here, particularly in glycerophospholipid pathways, may represent downstream consequences of reduced adiposity and improved metabolic health. Alternatively, they could act as contributing mechanisms or mediators linking the reduction in body weight to the normalization of pubertal timing. Disentangling the direct effects of EGCG on lipid metabolism from its overall anti-obesity effects will require future studies using pair-fed controls or other sophisticated experimental designs.

The EGCG dose used in this mouse study (2 mg/mL in drinking water) was effective in preventing obesity-induced precocious puberty. To assess its translational potential, we converted this dose to a human equivalent dose (HED) based on body surface area normalization (61). The calculated HED for a 30-kg child is approximately 960 mg EGCG per day. This dose is higher than the typical intake from dietary sources in our previous clinical trial (18). The safety of long-term EGCG supplementation, particularly in children, is a paramount concern. The European Food Safety Authority has suggested that daily EGCG intakes from supplements should not exceed 800 mg to minimize the risk of hepatotoxicity (62). The dose calculated from our model exceeds this guideline, highlighting that our study primarily serves as a proof-of-concept to elucidate mechanisms. Any potential future prophylactic use of EGCG for precocious puberty would necessitate extensive safety studies and careful dose optimization in pediatric populations. Future research should focus on identifying the lowest effective dose, potentially through the consumption of green tea itself or standardized extracts with known safety profiles, to maximize benefit while minimizing any potential risk.

Although the present study identified potential mechanisms through which EGCG may prevent obesity-induced precocious puberty, several limitations must be acknowledged. First, the research primarily focused on serum metabolomics and did not comprehensively investigate metabolic changes within key target organs—such as the hypothalamus, pituitary gland, ovaries, or adipose tissue—which are critically involved in the pathogenesis of this condition. Second, while the metabolomic analysis highlighted significant alterations in the glycerophospholipid metabolic pathway and molecular docking provided preliminary predictions, the study did not directly validate changes in the expression or activity of related enzymes (PISD, PLD, or PTDSS) using molecular biology techniques such as Western blot, qPCR, or enzyme activity assays. Consequently, the mechanistic evidence remains incomplete and primarily hypothetical. Future studies should include experimental validation of these potential targets. For instance, surface plasmon resonance or isothermal titration calorimetry could be used to directly measure binding affinity; enzyme activity assays could confirm functional inhibition or activation; and genetic approaches (e.g., knockdown or overexpression) in cellular models could assess the functional relevance of these targets in mediating EGCG’s effects. Third, the sample size (*n* = 5 per group), while comparable to some preliminary metabolomics studies, is relatively small. This may limit the statistical power and the generalizability of our findings. Future studies with larger cohorts are warranted to confirm and extend our observations. Fourth, a limitation of the present study is that we did not directly measure the concentration of EGCG in the serum or tissues of our mice to confirm its bioavailability. Nevertheless, extensive pharmacokinetic studies have demonstrated that orally administered EGCG is absorbed and can be detected in its intact form in the plasma and various organs of rodents at concentrations that are theoretically sufficient to support the high-affinity interactions predicted by our docking models (63–65). Future studies should directly quantify tissue EGCG levels and validate these putative interactions through *in vitro* binding assays and functional enzymatic studies.

Overall, the present study advances our understanding beyond the correlations established in our previous study (23, 24). While those studies highlighted alterations in the glycerophospholipid metabolism pathway at the systemic and tissue levels, the novel contribution here is the identification of PISD, PLD, and PTDSS as high-affinity potential direct targets of EGCG. This computational evidence provides a plausible mechanistic hypothesis for how EGCG reshapes lipid metabolism: through the direct modulation of these key enzymes. This represents a significant refinement of the mechanism of action and offers more precise targets for future experimental validation.

## 5 Conclusion

EGCG prevents obesity-induced precocious puberty by reshaping lipid metabolism, with key enzymes (PISD, PLD, and PTDSS) in glycerophospholipid metabolism serving as potential therapeutic targets. These findings provide a foundational hypothesis for further mechanistic investigation.

## Data Availability

The datasets presented in this study can be found in online repositories. The names of the repository/repositories and accession number(s) can be found in the article/Supplementary material.

## Footnotes

^1^ https://www.hmdb.ca

^2^ https://metlin.scripps.edu

^3^ https://cloud.majorbio.com

^4^ https://pubchem.ncbi.nlm.nih.gov
