# Supplementary material for: Exploring the molecular mechanism of EGCG in preventing obesity-induced precocious puberty based on serum metabolomics and molecular docking
Source: Front Nutr. 2025 Nov 6;12:1675535. doi: 10.3389/fnut.2025.1675535 (PMC12631206; doi:10.3389/fnut.2025.1675535)
Supplement: Supplementary file 1 [file Image_1.pdf]

Supplementary Files

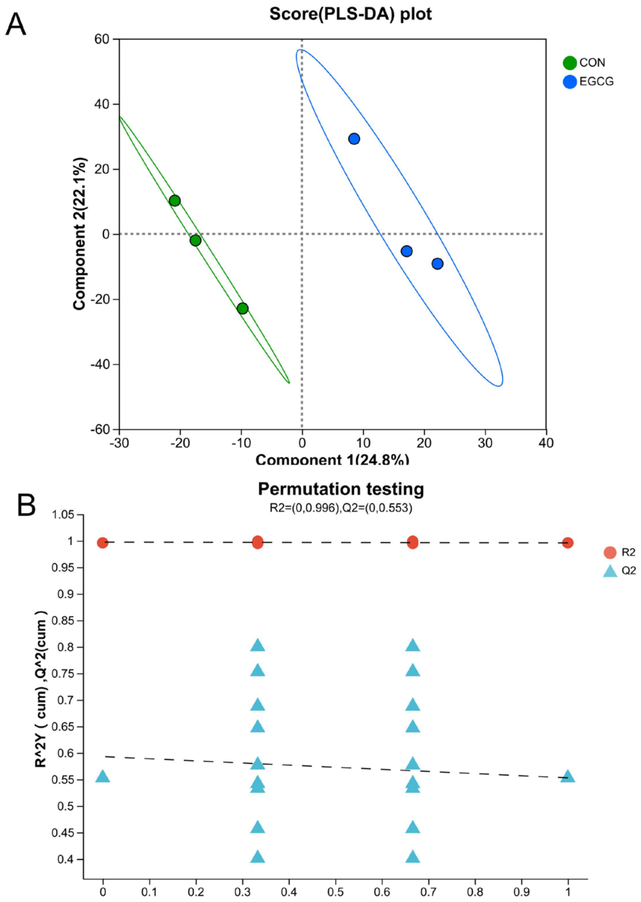

Figure S1. PLS-DA analysis comparing the serum metabolomes of the CON and EGCG groups.

Score plot shows minimal separation between the control group (CON, normal diet, green circles) and the group receiving EGCG supplementation on a normal diet (EGCG, blue circles). The explained variances for Component 1 and 2 were 24.8% and 22.1%, respectively. The lack of clear clustering, supported by poor model parameters (R^2Y=0.996, Q^2 =0.553), indicates that EGCG supplementation alone, in the absence of a high-fat diet, has a negligible impact on the serum metabolic profile.

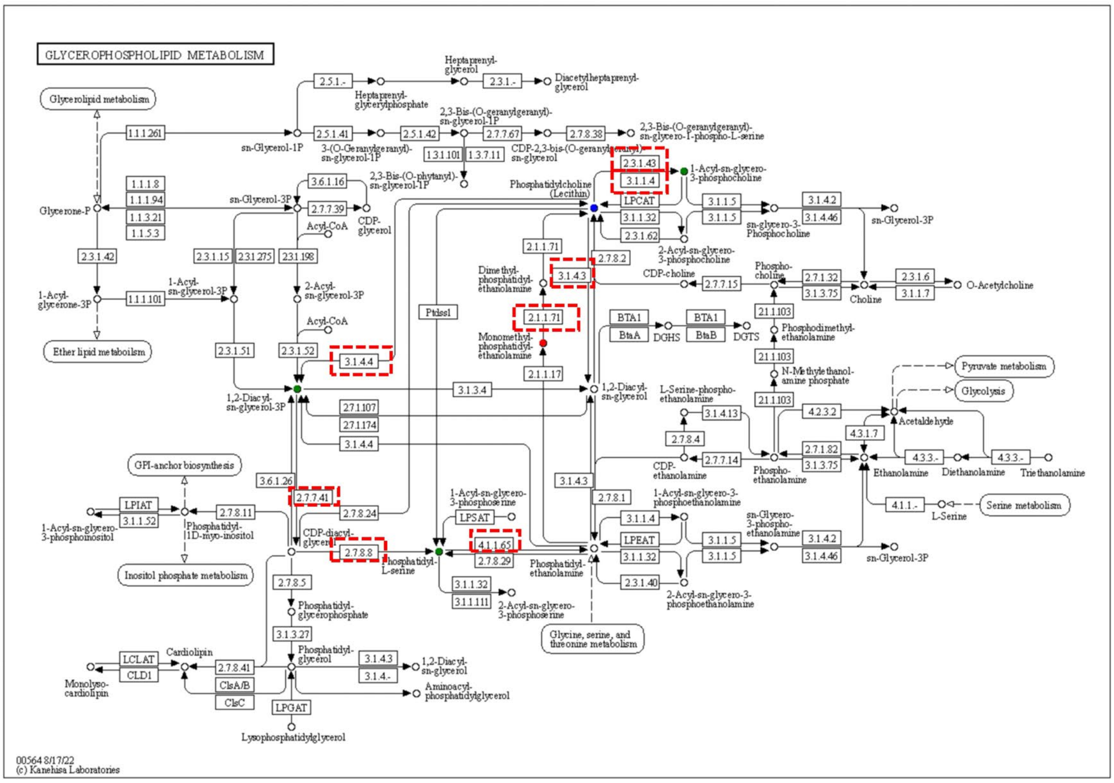

Figure S2. Glycerophospholipid metabolism pathway highlighting differential metabolites and key regulatory enzymes

Pathway map of glycerophospholipid metabolism. Differential metabolites are marked as follows: red boxes indicate up-regulated metabolites, green boxes indicate down-regulated metabolites, and blue boxes indicate metabolites showing both up- and down-regulation. The eight red dashed boxes highlight key upstream catalytic enzymes or downstream regulatory targets that directly control the differential metabolite levels of eight glycerophospholipid-related metabolites. EC 2.3.1.23(1-acylglycerophosphocholine O-acyltransferase), EC 3.1.1.4 (Phospholipase A2), EC 3.1.4.4 (Phospholipase D), EC 3.1.4.3 (Phospholipase C), EC 4.1.1.65 (Phosphatidylserine decarboxylase), EC 2.7.8.8 (Phosphatidylserine synthase), EC 2.1.1.71 (Phosphatidyl-N-methylethanolamine N-methyltransferase), and EC 2.7.7.41(CDP-diacylglycerol synthase).
